# Trapped lipopolysaccharide and LptD intermediates reveal lipopolysaccharide translocation steps across the *Escherichia coli* outer membrane

**DOI:** 10.1038/srep11883

**Published:** 2015-07-07

**Authors:** Xuejun Li, Yinghong Gu, Haohao Dong, Wenjian Wang, Changjiang Dong

**Affiliations:** 1 Biomedical Research Centre, Norwich Medical School, University of East Anglia, Norwich Research Park, Norwich, NR4 7TJ, UK; 2 Biomedical Sciences Research Complex, School of Chemistry, University of St Andrews, North Haugh, St Andrews, KY16 9ST, UK; 3 Laboratory of Department of Surgery, the First Affiliated Hospital, Sun Yat-sen University, 58 Zhongshan Road II, Guangzhou, Guangdong, 510080, China

## Abstract

Lipopolysaccharide (LPS) is a main component of the outer membrane of Gram-negative bacteria, which is essential for the vitality of most Gram-negative bacteria and plays a critical role for drug resistance. LptD/E complex forms a N-terminal LPS transport slide, a hydrophobic intramembrane hole and the hydrophilic channel of the barrel, for LPS transport, lipid A insertion and core oligosaccharide and O-antigen polysaccharide translocation, respectively. However, there is no direct evidence to confirm that LptD/E transports LPS from the periplasm to the external leaflet of the outer membrane. By replacing LptD residues with an unnatural amino acid *p*-benzoyl-L-phenyalanine (pBPA) and UV-photo-cross-linking in *E.coli*, the translocon and LPS intermediates were obtained at the N-terminal domain, the intramembrane hole, the lumenal gate, the lumen of LptD channel, and the extracellular loop 1 and 4, providing the first direct evidence and “snapshots” to reveal LPS translocation steps across the outer membrane.

All Gram-negative bacteria have an asymmetric bilayer outer membrane with lipopolysaccharide (LPS) forming the outer leaflet and phospholipid composing the inner leaflet^1^,^2^, respectively. Brought together by cations, lipopolysaccharide molecules form a permanent barrier, which is essential for the vitality of most Gram-negative bacteria and plays an important role for drug resistance^3^. LPS is an amphipathic macromolecule, normally containing three components, lipid A, core oligosaccharide and polysaccharide called O-antigen (Fig. 1a). Lipid A linked to its core oligosaccharide and the bactoprenyl-O-antigen units are each synthesized separately in the cytoplasm and transported by MsbA and Wzx, respectively, to the periplasmic side of the inner membrane, where the O-antigen is then polymerized and ligated en bloc to the lipid A core oligosaccharide terminal end, thus forming mature LPS molecules^1^.

Seven proteins, namely LptA, B, C, D, E, F and G, forming a trans-envelope complex, are responsible for transporting LPS from the inner membrane to the outer membrane^4^,^5^,^6^. LptBFG form a membrane complex transporter that recognizes and extracts LPS from the inner membrane and transports it to membrane protein LptC^7^,^8^,^9^,^10^, whereas LptC, LptA and the N-terminal domain of LptD, each possess a beta-jellyroll domain that assembles into a continuous LPS transport slide, which winds lipid A along its hydrophobic groove during LPS passage through the aqueous periplasm^11^,^12^,^13^,^14^. LptD forms a 26 β-stranded barrel, while LptE forms a plug located inside the LptD barrel to constitute an outer membrane LPS translocon^15^,^16^,^17^(Fig. 1b and Supplementary Fig. 1). The barrel is inserted into the outer membrane and consists of a hydrophilic channel sealed by the extracellular loops of LptD. The N-terminal domain of LptD directly delivers lipid A into the hydrophobic intramembrane hole of LptD, where it is inserted into the hydrophobic bilayer of the outer membrane, while the hydrophilic core oligosaccharide and O-antigen polysaccharide are translocated through the channel formed by LptD and LptE^18^. The lateral opening between strands β1C and β26C is required for LPS insertion and translocation^15^(For consistency with previous publications, we named the LptD N-terminal β-strands as β1-11a/bN, and the C-terminal domain β strands as β1-26C). However, there is no direct evidence to confirm that LPS is translocated across the outer membrane by the LptD/E translocon.

To monitor macromolecules passing through the outer membrane in bacteria is important and challenging work. Particularly, it is well known that hydrophobic compounds are difficult to go through the outer membrane. Although several outer membrane proteins have been reported to adopt lateral openings to transport hydrophobic compounds^19^,^20^,^21^,^22^, there is no direct evidence to reveal the transport or diffusion processes. We applied an *in vivo* approach to capture LPS intermediates within LptD in *E.coli* by using unnatural amino acid *p*-benzoyl-L-phenyalanine (pBPA) substitution and UV photo-cross-linking. The pBPA can be introduced into proteins at desired positions by mutating their amino acid codons to TAG (the amber codon), which will direct the incorporation of pBPA by the action of the orthogonal suppressor tRNA and aminoacyl-tRNA synthetase of *Methanococcus jannaschii*^23^,^24^,^25^. The carbonyl oxygen of pBPA can cross-link to any carbon-hydrogen bonds of molecules within 3 angstrom, when irradiated with UV light at 365 nm^26^. This method has been successfully used to trap the intermediates of LPS with LptA and LptC^27^, and of Wza with polysaccharide^28^, as well as the protein/protein complexes of SecY/SecA^29^, and LolC/LolA^30^. Here we report the detection of LptD-LPS intermediates trapped in *E.coli* cells, which provides the first direct evidence that LPS is transported within LptD and translocated across the outer membrane. These ‘snapshots’ highlight discrete LPS transport steps within LptD.

## Results

### Selection of LptD residues for pBPA incorporation

**Figure 1:**
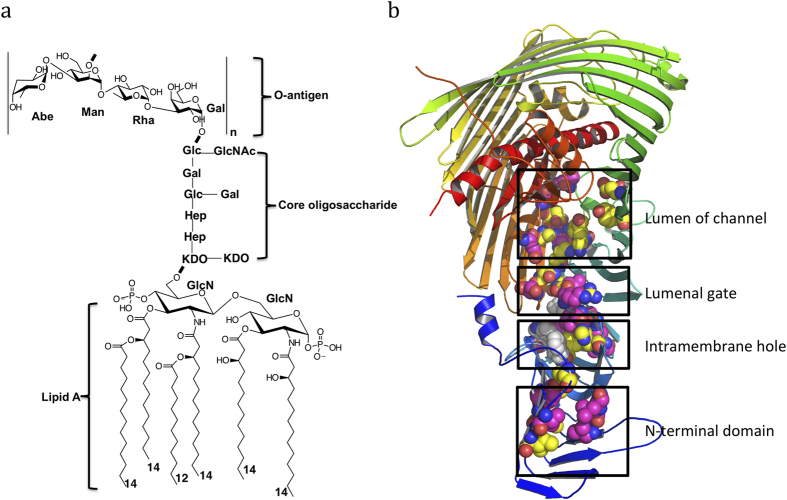
Lipopolysaccharide and its outer membrane translocon LptD/E. **a** lipopolysaccharide of *S. typhimurium* strain LT2. n = 4–40. LPS contains lipid A, core oligosaccharide and O-antigen. **b** LptD/E complex structure of *S. typhimurium* strain LT2. The N-terminal domain of *Salmonella* LptD is generated by modeling, based on the structure of *Shigella flexneri* LptD. The positions of residues are detected the cross-linking with LPS are shown in magentas, otherwise in yellow. The residues are selected at the N-terminal domain, the hydrophobic intramembrane hole, the lumenal gate and the lumen of barrel.

The structural and functional studies of LptD/E suggest that the N-terminal domain of LptD forms a slide with LptA and LptC, where it may use a similar mechanism to transport LPS^17^,^27^. To test this hypothesis, we selected 9 residues at the N-terminal domain for incorporation of pBPA (see text below, Fig. 1b and supplementary Fig. 1). All these residues are located along the hydrophobic groove of the N-terminal domain with their side chains pointing to the core of the jellyroll structure. Molecular dynamics simulations and functional assays have identified the intramembrane hole within LptD, which we suggest is where the lipid A of lipopolysaccharide is directly inserted into the hydrophobic bilayer of the outer membrane^18^. To prove this, we chose 11 residues for the mutations around the hydrophobic hole (Fig. 1b and text below).

The O-antigen and core oligosaccharide are hydrophilic, and suggested to be translocated through the LptD barrel^15^,^17^. The lumenal loop 1 and 2 form a lumenal gate, which is essential for the entry of core oligosaccharide and O-antigen into the LptD barrel^18^. Three residues at the lumenal loop 1 and 2 were chosen to check whether LPS goes through the lumenal gate (Fig. 1b and text below). To confirm whether the O-antigen and core oligosaccharide are translocated through the LptD channel, 11 residues located in the lumen of the channel were selected for the incorporation of pBPA and UV-crosslinking (Fig. 1b and text below). The extracellular loops will open the LptD pore, which will allow LPS to emerge to the bacterial surface^15^. To test this hypothesis, 3 residues on the extracellular L1 and 2 residues on L4 were chosen for cross-linking (see text below and Fig. 1b).

### Incorporation of pBPA into LptD

**Figure 2:**
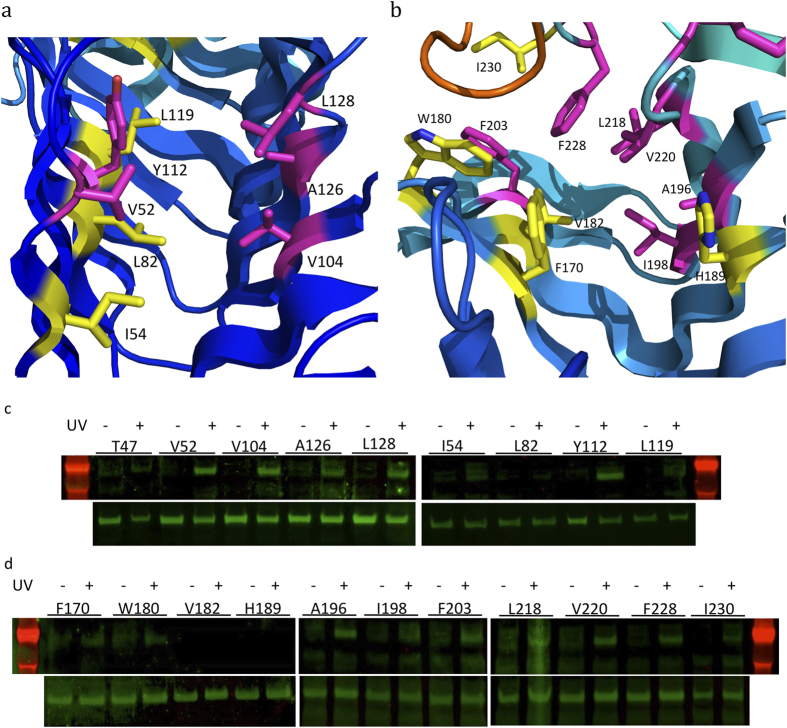
Detection of LptD and LPS intermediates at the N-terminal domain and the hydrophobic intramembrane hole. The positions of residues where cross-linking with LPS was detected are shown in magenta; residue positions with no LPS cross-linking are shown in yellow. **a** residues for incorporating pBPA at the N-terminal domain. **b** residues are selected for the incorporation of pBPA at the intramembrane hole. **c** the top lanes are western blots for detecting LptD and LPS complexes at the N-terminal domain, while the bottom lanes are the protein expression levels of the LptD variants. LptD/LPS complexes were observed at residues V52, V104, A126, L128 and Y112. **d** the top lanes are the detection of the LptD and LPS complexes at the intramembrane hole, and the bottom lanes are the protein expression levels of the LptD variants. The protein and LPS complexes are captured at residues A196, I198, F203, L218, V220 and F228.

**Figure 3:**
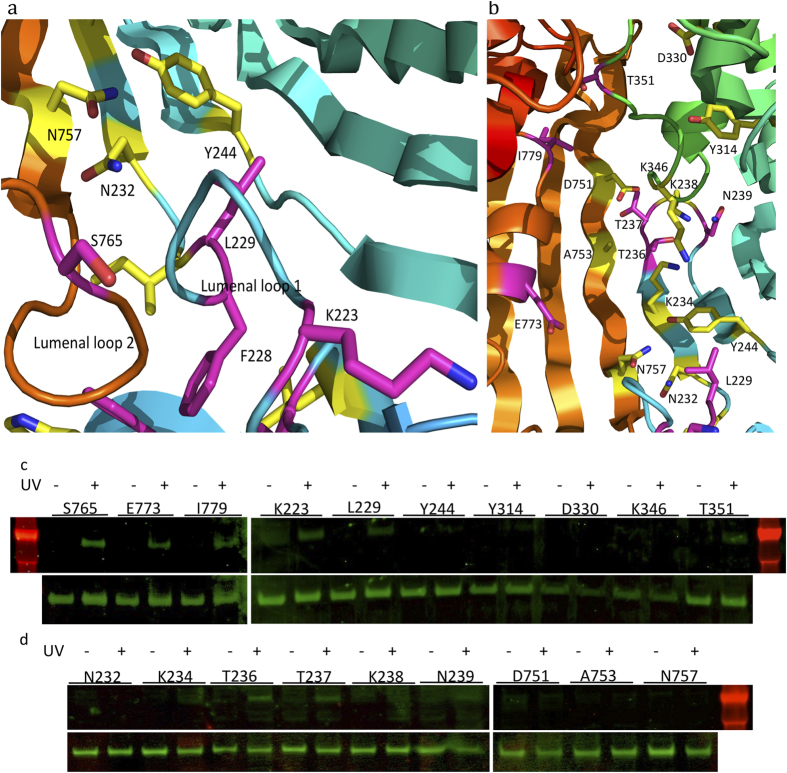
Observation of LptD and LPS complexes at the luminal gate, lumen of the barrel and the extracellular loops. The positions of residues where cross-linking with LPS was detected are shown in magenta; residue positions with no LPS cross-linking are shown in yellow. **a** the residues for the incorporation of pBPA at the lumenal gate. **b** the residues for incorporating pBPA at the lumen of the LptD barrel and the extracellular loops. **c** and **d** the top lanes are the detection of the LptD and LPS complexes at the lumenal gate, lumen of the barrel and the extracellular loops, and the bottom lanes are the protein expression levels of the LptD variants. The LptD/LPS complexes were detected at residues K223, L229 and S765 of the lumenal gate, T236, E773, I779 of the lumen of the LptD barrel, T236, N239 and T351 of the extracellular loops 1 and 4.

The above 39 residues of LptD at various locations along the N-terminal domain, the intramembrane hole, the lumenal gate, the barrel’s lumen, and the extracellular loops of LptD were selected for capturing “snapshots” of LPS transport within LptD and across the outer membrane. Mutagenesis was carried out on plasmid pCDFDuet-HisSenLptD which bears the *lptD* gene from *Salmonella typhimurium LT2*, using the primers listed in Supplementary Table 1. Overexpression of mutant LptDs was carried out in E. coli BL21(DE3) co-harbouring the pEVOL-pBpF plasmid, which provides the orthogonal tRNA and aminoacyl-tRNA synthetase. Both full-length and truncated LptDs were His-tagged at their N-termini and could be detected with a His tag antibody^15^. In the presence of pBPA, the amber codon within the mutant *lptD* gene was suppressed and the full-length LptD variants were observed (Figs 2, 3), where their protein expression levels were very similar. In contrast, no full length LptD was observed in the absence of pBPA (Supplementary Fig. 2).

To check whether these variant LptDs are functional *in vivo*, all the mutant *lptD* genes were introduced into the *lptD* depleted strain AM661 along with pEVOL-pBpF. As shown in Supplementary Fig. 2, in the presence of pBPA and absence of arabinose, AM661 harbouring pEVOL-pBpF and pCDFDuet-HisSenLptD-mutants (except for LptD-W180X) grew similarly well to cells containing plasmid encoding wild type LptD. On the other hand, in the absence of pBPA, AM661 harbouring pEVOL-pBpF and pCDFDuet-HisSenLptD-mutants didn’t grow, except for LptD-S765X and LptD-I779X (Supplementary Fig. 2), which only have a very short C-terminal segment truncated. These results are consistent with previous reports^15^,^18^. Given that W180 is the most conserved residue in LptD, it is not surprising that the W180 variant was not functional.

### LPS transport through the hydrophobic core of the N-terminal domain

The *in vivo* cross-linking of LPS to LptC and LptA with unnatural amino acid identified that LPS adducts could form at residues T32, I36, F95, Y114 and L116 of LptA and residues T47, F78, A172 and Y182 of LptC^27^. These residues are located inside of the jellyroll structures, suggesting that the hydrophobic grooves are involved in transporting LPS. To check whether the N-terminal domain of LptD uses a similar way to transport LPS, we replaced residues T47, V52, I54, L82, V104, Y112, L119, L128 and A126 with pBPA. After UV-radiation, LPS adducts were observed at residues V52, V104, Y112, A126 and L128 (Fig. 2a,c), all of which are located in the core at both sides of the jellyroll structure, suggesting that LPS is transferred through the LptD N-terminal domain along the hydrophobic groove, as similarly observed for LptA and LptC.

### Lipid A of LPS is inserted into the intramembrane hole

Molecules of the detergents LDAO and C8E4, which are mimics of lipid A, are bound to the LptD N-terminal domain core, pointing towards the intramembrane hole^17^,^18^. To confirm whether lipid A is inserted into the outer membrane via the intramembrane hole, we incorporated pBPA into LptD at residues F170, W180, V182, H189, A196, I198, F203, L218, V220, F228 and I230 around the hydrophobic intramembrane hole (Fig. 2b). No LPS adducts were observed without UV irradiation. However, after UV irradiation, LPS adducts were observed at positions A196, I198, F203, L218, V220 and F228 (Fig. 2d), which provide strong evidence that lipid A is inserted into the outer membrane via the intramembrane hole.

### LPS intermediates are captured at the lumenal gate

There are two lumenal loops at the bottom of the LptD barrel forming the lumenal gate, which allows the entry of core oligosaccharide and O-antigen into the barrel^18^. Residues K223 and L229 at the lumenal loop 1 and S765 on the lumenal loop 2 were selected for incorporation of pBPA (Fig. 3a), and adducts of LPS and LptD were observed at all three positions (Fig. 3c,d), which suggests that LPS goes through this gate.

### LPS is transferred through the LptD channel

The lumen of LptD is hydrophilic, and LptD is proposed to use a similar mechanism as that of Wza or AlgE for translocating O-antigen polysaccharide and core oligosaccharide^31^,^32^. To confirm that they are translocated through the barrel of LptD, we selected residues N232, K234, T236, Y244, Y314, D330, D751, A753, N757, E773 and I779 which are located in the lumen of the LptD barrel and replaced them with pBPA (Fig. 3b–d), individually. After UV irradiation, LPS/LptD adducts were observed at residues T236, E773 and I779. T236 is located in the lumen of β1C of LptD (Fig. 3c,d). These results prove that LPS is transported through the LptD barrel.

### LPS passes the extracellular loops of LptD

The LptD pore is sealed by extracellular loops in the LptD/E complex structures of *Salmonella typhimurium LT2* and *Shigella flexneri*, and our molecular dynamics simulations suggest that the pore will open during LPS translocation across the barrel^15^,^18^. Residues T237, K238 and N239 at extracellular L1 and K346 and T351 at extracellular L4 were chosen for the incorporation of pBPA (Supplementary Fig. 3a,b, Fig. 3c,d), where LptD and LPS intermediates were obtained at T237, N239 and T351, suggesting that the extracellular loops are open and that LPS passes through the pore of LptD to emerge at the bacterial surface.

## Discussion

LPS is a macromolecule containing the hydrophobic group, lipid A, and hydrophilic groups, core oligosaccharide and the O-antigen. Therefore, it is a great challenge to transport LPS through the water-filled periplasm and LptD barrel. The LptD/E outer membrane translocon is suggested to be responsible for forming the periplasmic slide with LptA and LptC to transport LPS from the inner membrane to the outer membrane. We have provided the first direct evidence to reveal the LPS transport steps by capturing ‘snapshots’ of LPS intermediates with LptD at the N-terminal domain, the intramembrane hole, the lumenal gate, through the lumen of the barrel and the extracellular loops. These ‘snapshots’ highlight the LPS transport steps within LptD. Other outer membrane proteins adopt a lateral opening mechanism to diffuse hydrophobic molecules into the outer membrane^19^,^20^,^21^,^22^. To our knowledge, this is the first direct evidence to reveal that a hydrophobic molecule (lipid A) is inserted or diffuses into the membrane through a hydrophobic hole.

All Gram-negative bacteria have the LptD protein and are predicted to have the N-terminal periplasmic domain and the C-terminal domain, which forms the 26 β-stranded barrel, although the amino acid sequence and length of protein may vary. This research can help us with understanding the mechanism of LPS transport and insertion into the outer membrane through LptD in almost all Gram-negative bacteria.

We have trapped the LPS intermediates along the hydrophobic groove of the LptD N-terminal domain, around the hydrophobic intramembrane hole, the lumenal gate, the lumen of LptD barrel and the extracellular loops of LptD barrel, where 19 LptD and LPS intermediates were obtained out of 39 positions (Figs 2a–d, 3a–d). The N-terminal domain of LptD forms the slide with around four LptA molecules and one LptC molecule across the periplasm^18^. By incorporating pBPA and UV crosslinking, and *in vitro* assays of LPS transport from the inner membrane to LptA, it was revealed that LptC and LptA transport LPS using their hydrophobic cores and ATP hydrolysis is required for LPS transport from LptC to LptA^27^. We speculate that LPS transport from LptA to N-terminal LptD also requires energy from ATP hydrolysis. The LPS–LptD intermediates were detected in two sides of the hydrophobic core of the N-terminal domain, confirming that LPS is transported along the hydrophobic core. Lipid A is delivered to the hydrophobic intramembrane hole through the hydrophobic core of the LptD N-terminal domain and inserted into the outer membrane, while the core oligosaccharide and O-antigen pass the lumenal gate and go through the channel of LptD/E. The lateral opening between β1C and β26C is triggered^15^, while the extracellular loops including L4 and L1 open the pore of LptD^33^,^34^. The core oligosaccharide and O-antigen of LPS emerge to the outer surface of bacteria, aided by LptE^33^,^34^.

This *in vivo* UV-photo cross-linking method has been used to study protein-protein interactions of SecY and SecA^29^, and LolA and LolC^30^, as well as LPS transport in LptA and LptC^27^, and polysaccharide transport in Wza^28^. To induce UV-photo crosslinking, pBPA is needed not only to be placed at the right position, but also to be at right orientations to the substrate. To achieve the best results, optimization is required for both *in vivo* UV cross-linking and sample preparation.

In summary, we report the first direct evidence to reveal how LptD transports LPS by *in vivo* UV photo crosslinking. The 19 “snapshots” highlight LPS translocation steps across the outer membrane, within LptD. To our knowledge, this is the first report to reveal discrete transport steps within a membrane protein at different parts of the protein by using this method. LPS is a main component of the outer membrane and plays an essential role in drug resistance. This work will help us to understand how Gram-negative bacteria build up their outer membrane, which is a fundamental biological process.

## Experimental Procedures

### Bacterial strains

*In vivo* photocrosslinking was carried out in *E. coli* strain BL21(DE3) [F^–^ *ompT hsdS*_B_ (r_B_^–^ m_B_^–^) *gal dcm* (DE3)], while *lptD* knockout strain AM661 [AM604 Δ*lptD*::*kan/ araBp-lptD*]^35^,^36^ was used for functional analysis. Bacteria were grown at 37 °C in LB broth containing appropriate antibiotics (34 μg/ml chloramphenicol, 30 μg/ml kanamycin, and/or 50 μg/ml streptomycin).

### Plasmid construction

The *lpt*D gene of *S. typhimurium* strain LT2, encoding a 6xHis tag between Leu27 and Ala28^15^, was cloned into pCDFDuet-1 (Novagen) between the Nde I and Xho I sites to make plasmid pCDFDuet-HisSenLptD, upon which site-directed mutagenesis^37^ was carried out. The oligonucleotides used in this study are listed in Table S1. All the mutations were confirmed by sequencing.

### *In vivo* photo-crosslinking

Photo-crosslinking was carried out according to the published method^27^ with some modifications. Fifty ml LB broth containing 50 μg/ml streptomycin and 34 μg/ml chloramphenicol was inoculated with an overnight culture of BL21(DE3) harbouring pEVOL-pBpF^24^ and pCDFDuet-HisSenLptD (wild type or mutant), and was incubated at 37 °C for two hours. Overexpression of His-tagged LptD was induced with 16 μM isopropyl β-D-1-thiogalactopyranoside (IPTG), plus 0.5 mM pBPA (Bachem) and 0.02% arabinose, which helped to suppress the amber stop codon and incorporate the unnatural amino acid pBPA. The culture was grown to mid-log phase at 37 °C and half of the culture (25 ml) was irradiated with UV light at 365 nm for 5 min, while the other half was untreated. His-tagged LptD was extracted and purified according to Okuda’s method^27^ and analysed by Western blotting.

### Western blotting

Protein samples were heated at 90 °C for 5 min and separated on Invitrogen Bolt 4-12% Bis-Tris Plus gel. Western blotting was carried out with a Bio-Rad semi-dry device. Mouse monoclonal antibody against LPS core (from HyCult Biotechnology), or mouse monoclonal antibody against His-tag (from Novagen) were used as the primary antibody, while the secondary antibody was IRDye800CW goat-anti-mouse from LI-COR. The blot was scanned with the Odyssey Infrared Imaging System from LI-COR. All procedures were carried out as recommended by the manufacturers.

### Functional assays

*Salmonella lptD* mutants were used for functional assays in the *E. coli lptD* depleted strain, AM661. Plasmids with empty vector pCDFDuet-1, wild type *lptD* and its different variants were transformed into *E. coli* AM661, and then grown on LB agar plates supplemented with 0.5 mM *p*-benzoyl-L-phenyalanine (pBPA) and antibiotics (30 μg ml^−1^ kanamycin, 50 μg ml^−1^ streptomycin and 34 μg ml^−1^ chloramphenicol). For pCDFDuet-1, 0.2% L-arabinose was also included. A single-colony was inoculated into 5 mL LB medium supplemented with antibiotics before streaking onto LB agar plates supplemented with antibiotics, with or without 0.5 mM pBPA.

## Additional Information

**How to cite this article**: Li, X. *et al*. Trapped lipopolysaccharide and LptD intermediates reveal lipopolysaccharide translocation steps across the *Escherichia coli* outer membrane. *Sci. Rep*. **5**, 11883; doi: 10.1038/srep11883 (2015).

## Supplementary Material

Supplementary Information
