# Supplementary material for: Trapped lipopolysaccharide and LptD intermediates reveal lipopolysaccharide translocation steps across the Escherichia coli outer membrane
Source: Sci Rep. 2015 Jul 7;5:11883. doi: 10.1038/srep11883 (PMC4493717; doi:10.1038/srep11883)
Supplement: Supplementary Information [file srep11883-s1.pdf]

Supplementary Data

Trapped lipopolysaccharide and LptD intermediates reveal lipopolysaccharide translocation steps across the *Escherichia coli* outer membrane

Xuejun Li[1], Yinghong Gu[1], Haohao Dong[2], Wenjian Wang[3] and Changjiang Dong[1*]

[1]Biomedical Research Centre, Norwich Medical School, University of East Anglia, Norwich Research Park, Norwich, NR4 7TJ, UK;

[2]Biomedical Sciences Research Complex, School of Chemistry, University of St Andrews, North Haugh, St Andrews, KY16 9ST, UK;

[3]Laboratory of Department of Surgery, the First Affiliated Hospital, Sun Yat-sen University, 58 Zhongshan Road II, Guangzhou, Guangdong, 510080, China.

Table S1: oligonucleotides used in this study

Names	Sequences
T47Xfor	GGCGATTAGAACGATCTGCCGGTTACTATCAATG
T47Xbac	GATCGTTCTAATCGCCTTTTACCAGAGGACGATCGTAGC
V52Xfor	CTGCCGTAGACTATCAATGCCGATAACGCTAAAG
V52Xbac	TTGATAGTCTACGGCAGATCGTTGGTATCGCCT
I54Xfor	GGTTACTTAGAATGCCGATAACGCTAAAGGTAACTAC
I54Xbac	CGGCATTCTAAGTAACCGGCAGATCGTTGGTATCG
L82Xfor	AGCCGCTAGCAAGCGGATGAAGTGCAGCTTCATCAGA
L82Xbac	CGCTTGCTAGCGGCTATTCCCCTGCATAATGTCCA
V104Xfor	CGCACCTAGGATGCGCTGGGTAATGTGCATTATGA
V104Xbac	CGCATCCTAGGTGCGTACAGGTTCCGGCTGAC
Y112Xfor	ATGTGCATTAGGATGACAATCAGGTCATCCTTAAAGGG
Y112Xbac	TTGTCATCCTAATGCACATTACCCAGCGCATCGACG
L119Xfor	GGTCATCTAGAAAGGGCCGAAGGGCTGGGCGAA
L119Xbac	GCCCTTTCTAGATGACCTGATTGTCATCATAATGCAC
A126Xfor	GGCTGGTAGAACCTGAACACCAAAGACACGAACG
A126Xbac	TTCAGGTTCTACCAGCCCTTCGGCCCTTTAAGGA
L128Xfor	GCGAACTAGAACACCAAAGACACGAACGTCTGGGA
L128Xbac	TTGGTGTTCTAGTTCGCCCAGCCCTTCGGCCCTTTA
F170Xfor	GGCAGCTAGACCTCCTGTCTGCCTGGCTCCGAT
F170Xbac	GGAGGTCTAGCTGCCGTTTTCCAGAATGGTATAGCG
W180Xfor	CGATACCTAGAGCGTGGTGGGGAGTGAAGTCAT
W180Xbac	CACGCTCTAGGTATCGGAGCCAGGCAGACAGGA
V182Xfor	CTGGAGCTAGGTGGGGAGTGAAGTCATCCATGACCG
V182Xbac	CCCCACCTAGCTCCAGGTATCGGAGCCAGGCAG
H189Xfor	AGTCATCTAGGACCGCGAAGAACAGGTTGCGGAG
H189Xbac	GCGGTCCTAGCTAACTTCACTCCCCACCACGCTC
A196Xfor	ACAGGTTTAGGAGATCTGGAACGCCCGGTTTAAAG
A196Xbac	AGATCTCCTAAACCTGTTCTTCGCGGTCATGGAT
I198Xfor	GCGGAGTAGTGGAACGCCCGGTTTAAAGTAGGTC
I198Xbac	CGTTCCACTACTCCGCAACCTGTTCTTCGCGGTC
F203Xfor	GCCCGGTAGAAAGTAGGTCCGGTTCCGATCTTTTA
F203Xbac	CCTACTTTCTACCGGGCGTTCCAGATCTCCGCAA
L218Xfor	ATTTACAGTAGCCCGTCGGTGACAAGCGTCGCTC
L218Xbac	GACGGGCTACTGTAAATAGGGGCTATAAAAGATCG
V220Xfor	GCTACCCTAGGGTGACAAGCGTCGCTCAGGTT
V220Xbac	TGTCACCCTAGGGTAGCTGTAAATAGGGGCTATAAA
K223Xfor	CGGTGACTAGCGTCGCTCAGGTTTCCTGATCC
K223Xbac	GCGACGCTAGTCACCGACGGGTAGCTGTAAATA
F228for	CTCAGGTTAGCTGATCCCGAACGCGAAATACACG
F228bac	GGATCAGCTAACCTGAGCGACGCTTGTCACCGACG
L229for	CAGGTTTCTAGATCCCGAACGCGAAATACACGACCA
L229bac	CGGGATCTAGAAACCTGAGCGACGCTTGTCACC
I230for	GTTTCCTGTAGCCGAACGCGAAATACACGACCAAGAA
I230bac	GTTCGGCTACAGGAAACCTGAGCGACGCTTGTCA
N232for	GATCCCGTAGGCGAAATACACGACCAAGAACTATTTCG

N232bac	TATTTCGCCTACGGGATCAGGAAACCTGAGCGAC
K234for	AACGCGTAGTACACGACCAAGAACTATTTCGAGTTCTA
K234bac	TCGTGTACTACGCGTTCGGGATCAGGAAACCTG
T236for	CGAAATACTAGACCAAGAACTATTTCGAGTTCTACTTA
T236bac	TCTTGGTCTAGTATTTCGCGTTCGGGATCAGGAA
T237for	AATACACGTAGAAGAACTATTTCGAGTTCTACTTACCG
T237bac	ATAGTTCTTCTACGTGTATTTCGCGTTCGGGATCA
K238for	ACGACCTAGAACTATTTCGAGTTCTACTTACCGTATTA
K238bac	GAAATAGTTCTAGGTCGTGTATTTCGCGTTCGGGA
N239for	ACCAAGTAGTATTTCGAGTTCTACTTACCGTATTACTG
N239bac	CTCGAAATACTACTTGGTCGTGTATTTCGCGTTC
Y244for	CGAGTTCTAGTTACCGTATTACTGGAACATCGCGCC
Y244bac	ACGGTAACTAGAACTCGAAATAGTTCTTGGTCGTGTA
Y314Xfor	GGTTATTCTAGTGGCAGCACTCAGGCGTGATGGA
Y314Xbac	GCTGCCACTAGAATAACCAGCGGTGCTTATCGCCC
D330for	TTAACGTCTAGTACACCAAAGTCAGCGACTCCAGC
D330bac	TTGGTGTACTAGACGTTAAAACGCCACACCTGATCC
K346for	CGACAGTTAGTACGGTTCCAGTACCGACGGCTACG
K346bac	GAACCGTACTAACTGTCGAAATCGTTAAAGTAGCTGGAG
T351Xfor	TTCCAGTTAGGACGGCTACGCAACGCAGAAATTCA
T351Xbac	GCCGTCCTAACTGGAACCGTACTTACTGTCGAAA
D751for	CGATTTATTAGAACGCGATTGGCTTCAACATTGAG
D751bac	TCGCGTTCTAATAAATCGCGTGTTGTTTATCGTTATC
A753for	TTATGATAACTAGATTGGCTTCAACATTGAGCTGCG
A753bac	AGCCAATCTAGTTATCATAAATCGCGTGTTGTTTATC
N757for	GGCTTCTAGATTGAGCTGCGCGGTTTGAGCTC
N757bac	AGCTCAATCTAGAAGCCAATCGCGTTATCATAAATCG
S765Xfor	TTTGAGCTAGAACTACGGCCTCGGCACGCAAGAAA
S765Xbac	CCGTAGTTCTAGCTCAAACCGCGCAGCTCAATGT
E773for	CACGCAATAGATGTTGCGTTCGAACATTCTGCC
E773bac	CGCAACATCTATTGCGTGCCGAGGCCGTAGTTA
I779Xfor	CGAACTAGCTGCCGTACCAAAGCTCTATGTAACTC
I779Xbac	TACGGCAGCTAGTTCGAACGCAACATTTCTTGCGTG

a b

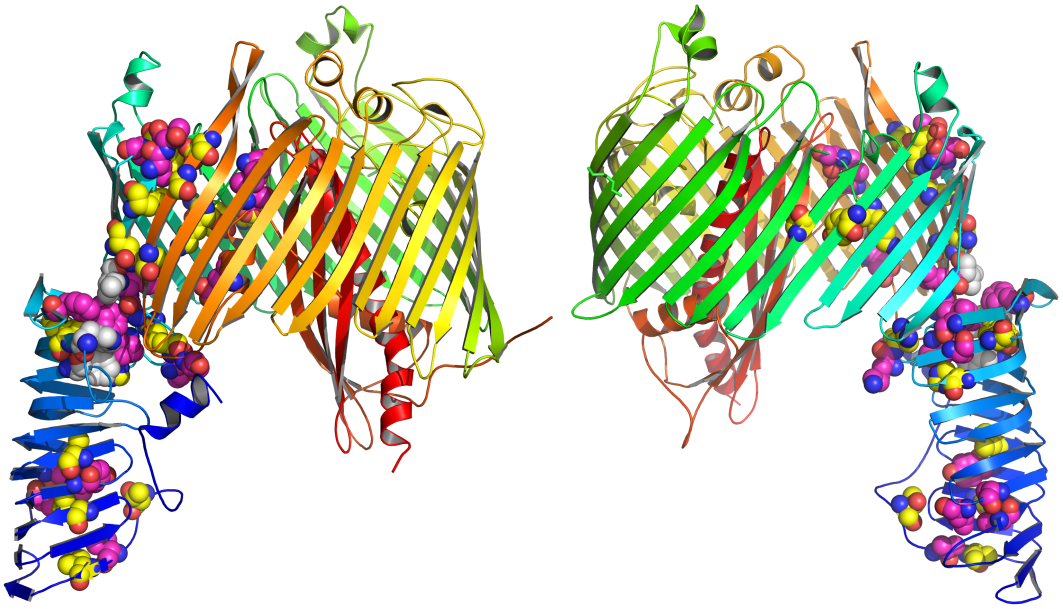

Figure S1| Residues of *S. typhimurium* LptD are selected for XpBPA incorporation and UV cross-linking. Residues in magenta can cross-link with LPS. Figure a rotates 180 degree along y-axis to become figure b.

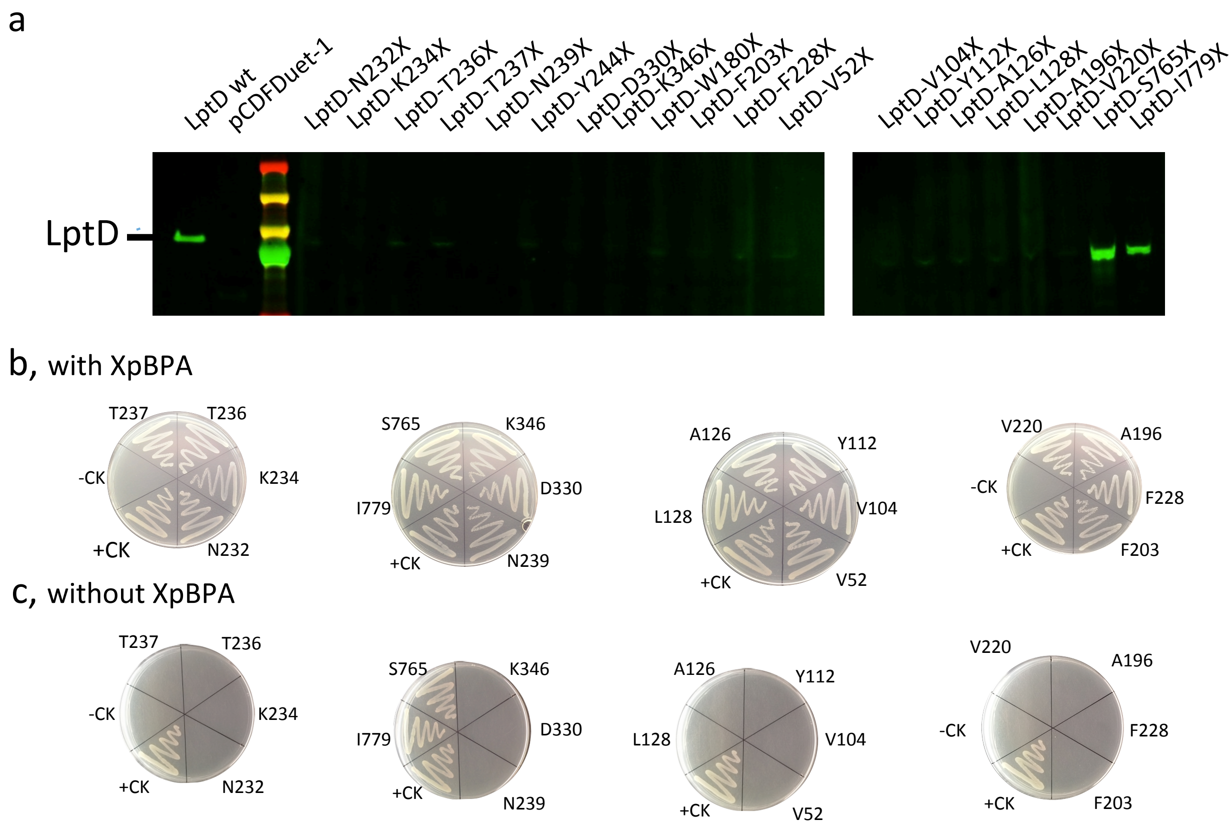

Figure S2 | Incorporation of pBPA into mutant LptDs could produce a functional LptD (only some examples are shown here). a, Total cell lysate from BL21(DE3) expressing His-tagged wild type or mutant LptDs were detected with anti-his antibody. Without pBPA, full-length LptDs couldn't be produced (in contrast to Fig2c, 2d, 3c and 3d, where full length LptD variants could be produced in the presence of pBPA). b, LptD depleted AM661 strain expressing mutant LptDs could grow similarly well to that expressing wild type LptD in the presence of pBPA. c, Except for S765 and I779, mutations at other positions resulted in the death of the host AM661 in the absence of pBPA. +CK, positive control (AM661 harbouring plasmid expressing wild type LptD); -CK, negative control (AM661 containing empty plasmid).

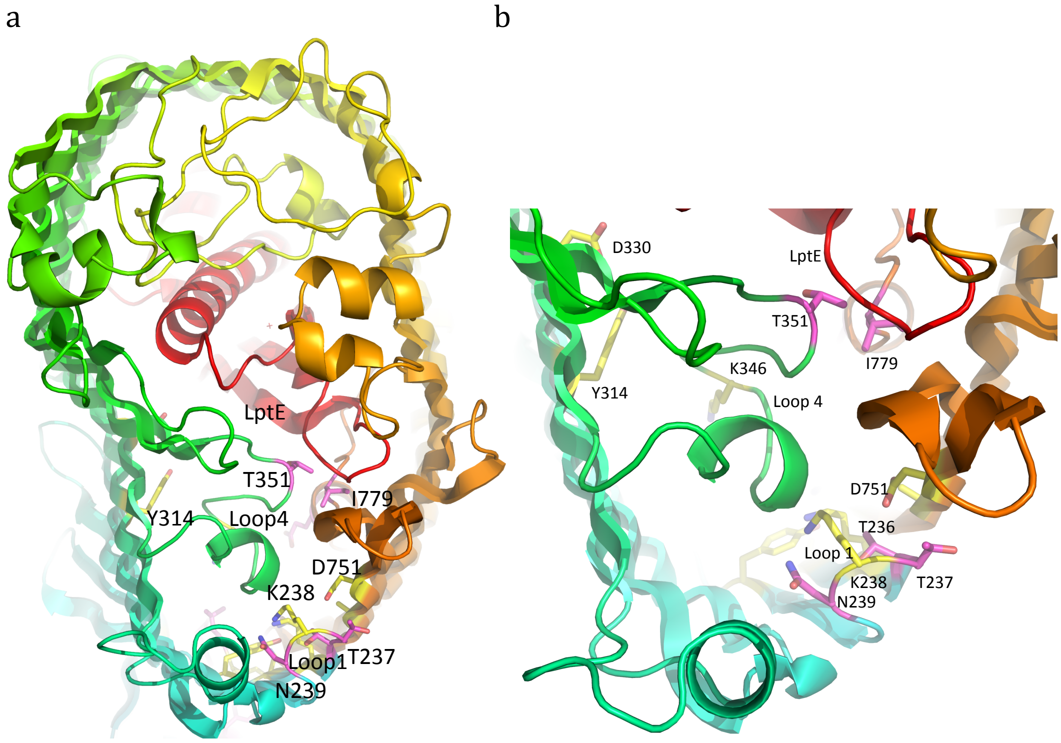

Figure S3| Extracellular view of LptD/E translocon. Residues in magenta can cross-link with LPS. Extracellular loop 1 and 4 open the pore for the core oligosaccharide and O-antigen exit from the LptD channel. Figure b is part of figure a.
